# Association Between Body Mass Index (BMI), Vitamin D, and Testosterone Levels

**DOI:** 10.7759/cureus.71509

**Published:** 2024-10-15

**Authors:** Mehwash Iftikhar, Nazir Shah, Imran Khan, Mian Mufarih Shah, Muhammad Numan Saleem

**Affiliations:** 1 Internal Medicine, Hayatabad Medical Complex Peshawar, Peshawar, PAK

**Keywords:** bmi, body mass index, obesity, testosterone, vitamin d

## Abstract

Introduction

Obesity is measured scientifically by calculating body mass index (BMI). Body mass index in men is linked to various hormonal imbalances. This study aims to observe the relationships between BMI, vitamin D, and testosterone levels in patients attending the outpatient clinic at Hayatabad Medical Complex, Peshawar, Pakistan.

Methods

This observational cross-sectional study involved 272 patients, presenting to the medical outpatient department from January 1^st^, 2023, to December 31^st^, 2023. Body mass index, serum vitamin D, and testosterone levels were measured for each participant. Body mass index was categorized into normal, overweight, and obese. Statistical analysis was calculated, including descriptive statistics, logistic regression, and correlation analysis to evaluate associations between these variables.

Results

The mean BMI of the participants was 25.82 ± 7.88 kg/m². A significant inverse correlation was observed between BMI and vitamin D levels (r = -0.79, p < 0.001) and between BMI and testosterone levels (r = -0.87, p < 0.001). A positive correlation was found between vitamin D and testosterone levels (r = 0.87, p < 0.001). Logistic regression analysis revealed that higher BMI in the range of overweight or above was associated with a 2.5-fold increase in the likelihood of vitamin D deficiency (odds ratio (OR) = 2.50, 95% CI = 1.8-3.5, p < 0.001) and a 3.1-fold increase in the likelihood of low testosterone levels (OR = 3.10, 95% CI = 2.2-4.3, p < 0.001).

Conclusion

In this study, higher BMI is significantly associated with lower vitamin D and testosterone levels. These findings suggest that addressing obesity could help mitigate hormonal imbalances, such as vitamin D deficiency and low testosterone, which are linked to metabolic health risks. It can also be hypothesized that obesity can be a risk factor for vitamin D and testosterone deficiency.

## Introduction

Obesity, defined scientifically as an individual’s body mass index (BMI) of >30 kg/m^2^, is a public health concern globally. It is associated with protean metabolic and hormonal imbalances.

Body mass index, a simple measure calculated by dividing the person’s weight in kilograms by the square of the height in meters, is used to categorize individuals into underweight, normal weight, and overweight or obese categories. According to the World Health Organization (WHO), a BMI of 18.5 to 24.9 kg/m^2^ is considered normal, while a BMI of 25.0 to 29.9 kg/m^2^ indicates overweight, and a BMI of 30 kg/m^2^ or higher classifies an individual as obese [1].

Vitamin D and testosterone levels are two critical biomarkers associated with BMI. Vitamin D, a fat-soluble vitamin, is crucial for innumerable body functions, including bone health, immune function, and even functions as diverse as sleep regulation and emotional health. Vitamin D has been linked to a wide array of diverse metabolic processes [2]. Deficiency in Vitamin D is prevalent in individuals with higher BMIs, as excess body fat is able to sequester Vitamin D, lowering its bioavailability [3]. Studies have indicated that low vitamin D levels may be inversely related to BMI, with obese individuals often showing deficient vitamin D levels [4].

Testosterone is an androgenic hormone that plays a crucial role in muscle mass, fat distribution, and overall metabolic health in both men and women. Research has suggested that testosterone levels decline with increasing BMI, particularly in men, contributing to conditions such as metabolic syndrome and hypogonadism [5]. The complex interaction between BMI, vitamin D, and testosterone has been the subject of numerous studies in the past, yet conclusive evidence regarding their interrelationship remains hypothetical [6].

Our study aims to explore the relationship between BMI, vitamin D, and testosterone levels in patients attending the outpatient medical clinic of the Hayatabad Medical Complex in Peshawar, Pakistan. We hypothesized that individuals with higher BMIs shall exhibit lower vitamin D and testosterone levels. In contrast, those with normal or low BMIs will show more variable or no relationships between these parameters.

## Materials and methods

Study design

This observational cross-sectional study was conducted at the medical outpatient department of the Hayatabad Medical Complex, a tertiary care hospital located in Peshawar, Pakistan. The study period spanned from 1^st^ January 2023 to 31^st^ December 2023. Ethical approval for the study was obtained from the ethical committee of the hospital before commencement ((Doc No. HMC-QAD-F-00; approval no. 2211).

A total of 272 patients were enrolled in the study. Inclusion criteria were male adults aged 18 years and older, with no known chronic conditions that might interfere with vitamin D or testosterone levels, such as chronic kidney disease or hypogonadism. All female patients were excluded from the study. Patients were excluded if they had been receiving vitamin D supplementation or testosterone replacement therapy within six months prior to the study.

Body mass index was calculated using the standard formula (weight in kilograms divided by height in meters squared). Based on BMI, participants were classified into one of three categories: normal BMI (18.5-24.9 kg/m^2^), overweight (25.0-29.9 kg/m^2^), or obese (≥30.0 kg/m^2^). Blood samples were collected from all participants after an overnight fast. Serum vitamin D and testosterone levels were measured using enzyme-linked immunosorbent assay (ELISA) kits, following the manufacturer’s protocols.

Serum vitamin D levels were measured in ng/mL, with levels below 20 ng/mL considered deficient. Testosterone levels were measured in ng/dL, with levels below 300 ng/dL considered low.

Statistical analysis

Data were analyzed using IBM SPSS Statistics software, version 26.0 (IBM Corp., Armonk, NY). Descriptive statistics were calculated for all variables, including mean, standard deviation, and confidence intervals. Logistic regression analysis was performed to assess the relationship between BMI, vitamin D, and testosterone levels. Pearson’s correlation coefficient was used to determine the strength and direction of the relationships between these variables. P-values less than 0.05 were considered statistically significant with a 95% confidence interval.

**Figure 1:**
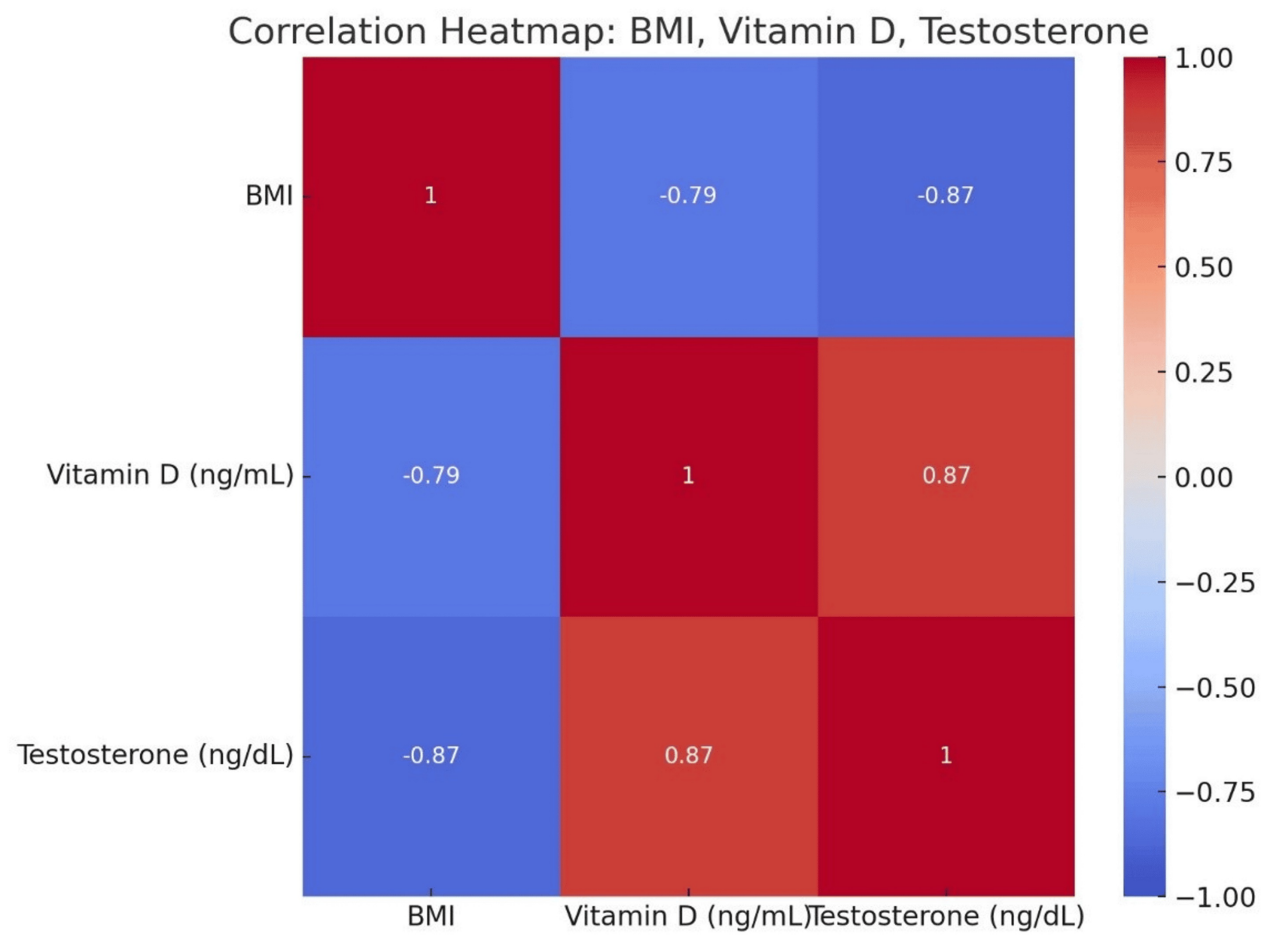
A correlation heatmap showing the relationship between BMI, vitamin D, and testosterone levels in the study participants

A correlation heat map of all three variables was constructed in Python software (Python Software Foundation, Fredericksburg, VA). The graphical representation of the three variables can be easily understood through a correlation heat map (Figure 1).

## Results

A total of 272 male participants were included in the study. The age range was 19 to 63 years, with a mean age of 45.6±10.3 years. The mean BMI of the participants was 25.82 ± 7.88 kg/m², ranging from 15.0 to 40.0. The mean vitamin D level was 23.36 ng/mL ± 8.49. Thirty-eight percent (38%) of participants displayed vitamin D deficiency (defined as levels below 20 ng/mL). Testosterone levels ranged from 201.5 to 692.4 ng/dL, with a mean of 387.8 ng/dL ± 133.33. Table 1 shows the demographic features of the study population. Table 2 shows the statistical description of BMI, vitamin D level, and testosterone level in the study patients.

**Table 1:** Demographic details of the participants

Participant characteristic	Detail
Total patients	272
Mean age (+_SD)	45.6(SD±10.3)
Gender	Male
Mean BMI (± SD)	25.816 ± 6.49 kg/m^2^

**Table 2:** Descriptive statistics of the study population

Statistics	BMI (kg/m^2^)	Vitamin D (ng/ml)	Testosterone (ng/dl)
Number of patients	272	272	272
Mean	25.816	23.357	387.797
Standard deviation	7.881	8.493	133.326
Range	15.0 - 40	10.0 - 39.8	201.5 - 692.4

Primary outcomes

Body Mass Index and Vitamin D Levels

**Figure 2:**
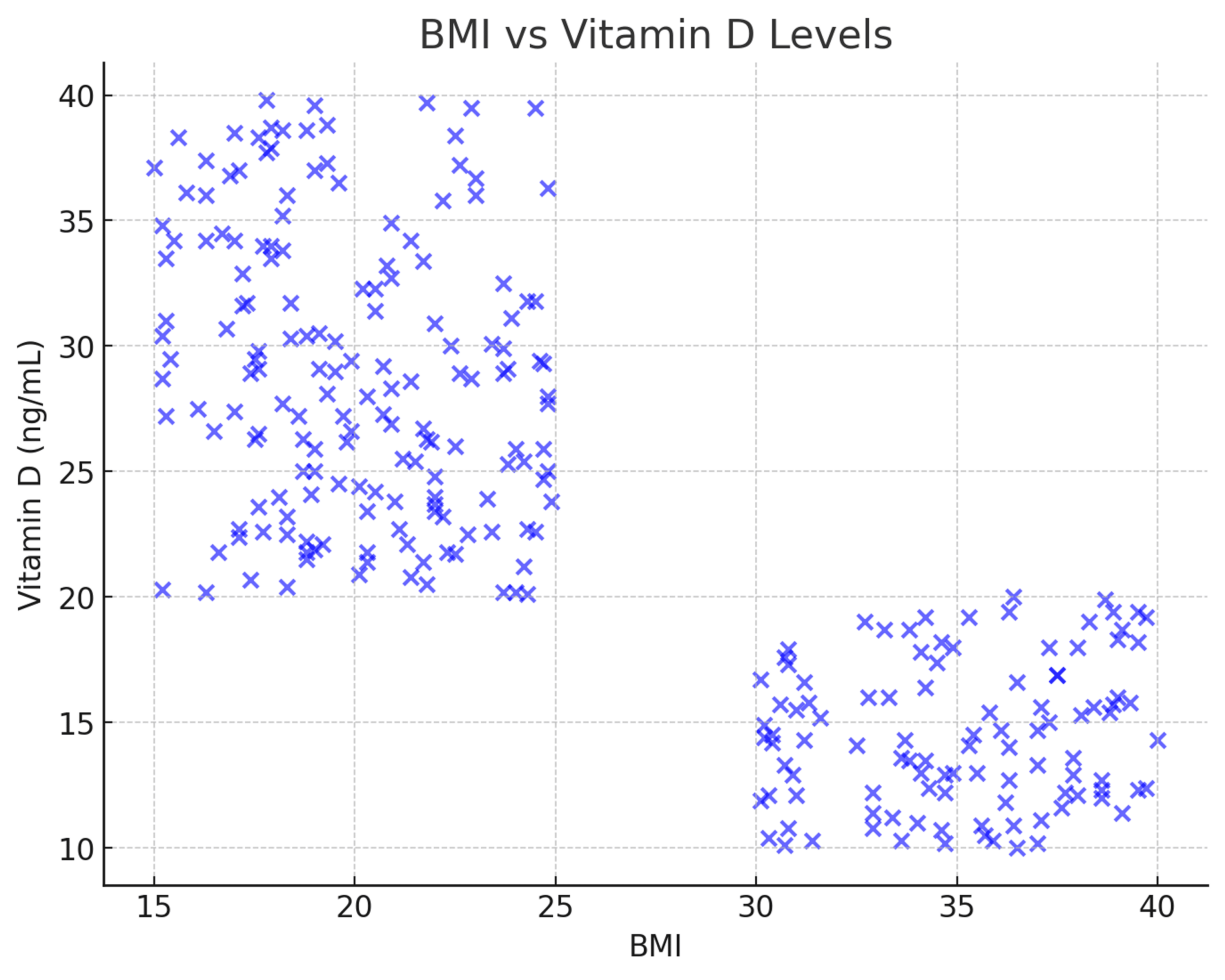
A scatter plot depicting the inverse correlation between BMI and vitamin D levels

A significant inverse correlation was observed between BMI and vitamin D levels (r = -0.79, p < 0.001). A high BMI is associated with low vitamin D levels. Among the participants classified as obese (BMI ≥ 30), 75% had vitamin D deficiency. Figure 2 illustrates the relationship between BMI and vitamin D levels.

Body Mass Index and Testosterone Levels

**Figure 3:**
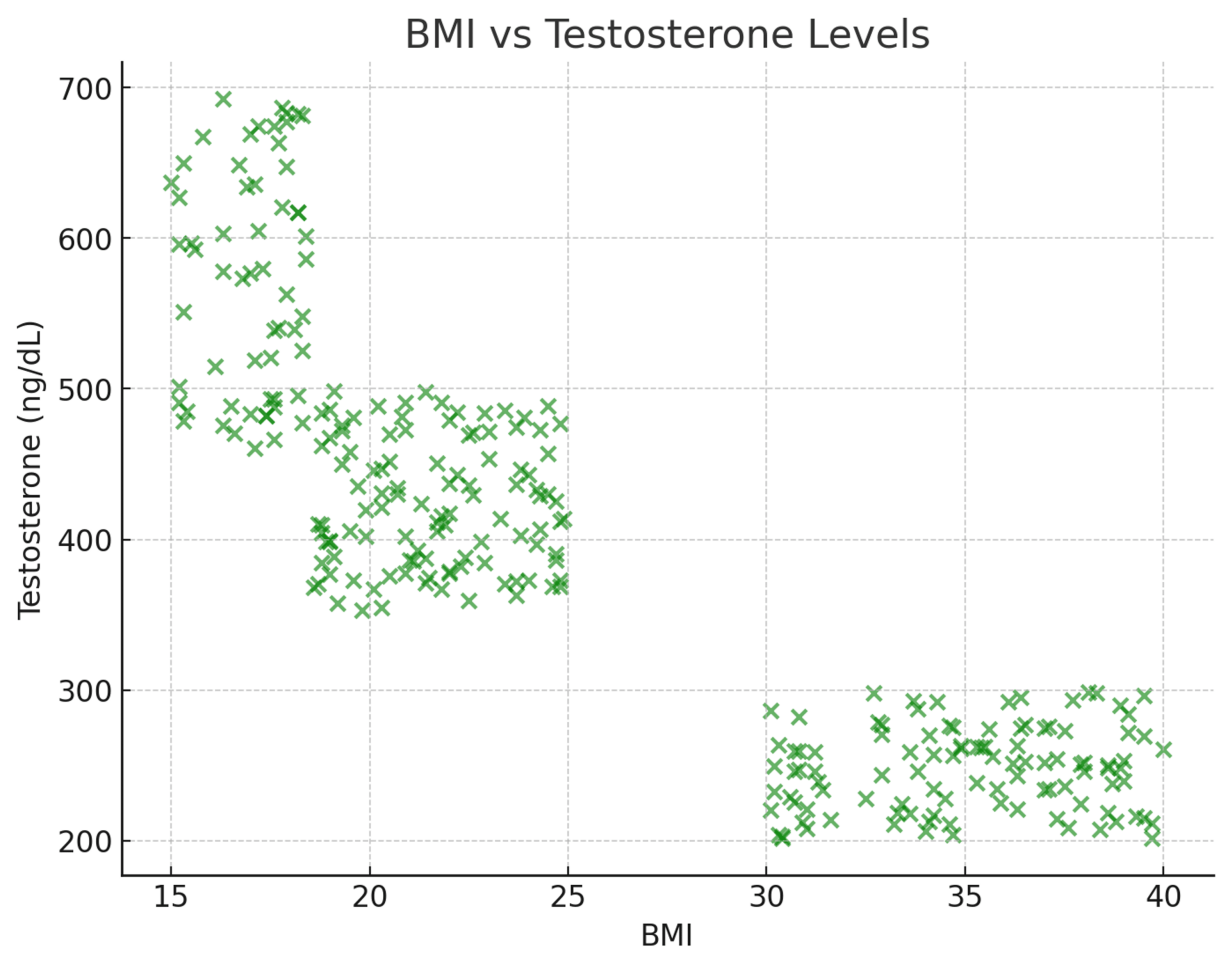
A scatter plot illustrating the inverse relationship between BMI and testosterone levels

A similar inverse relationship was found between BMI and testosterone levels (r = -0.87, p < 0.001). Testosterone levels decreased with increasing BMI, with obese participants exhibiting significantly lower testosterone levels compared to non-obese individuals. Figure 3 illustrates the relationship between BMI and testosterone levels.

Vitamin D and Testosterone

A strong positive correlation was observed between vitamin D and testosterone levels (r = 0.87, p < 0.001). Participants with lower vitamin D levels also had lower testosterone levels, suggesting a potential interrelationship between these two biomarkers. Figure 3 indicates the inverse relationship between BMI and the two study biomarkers. Table 3 shows the correlation of BMI with vitamin D and testosterone, along with the correlation of vitamin D and testosterone.

**Table 3:** Primary outcomes

Parameters	Correlation coefficient (r)	p-value	Interpretation
BMI and vitamin D levels	-0.79	0.001	Inverse correlation between BMI and vitamin D levels
BMI and testosterone levels	-0.87	0.001	Inverse correlation between BMI and testosterone levels
Vitamin D and testosterone levels	0.87	0.001	Positive correlation between vitamin D and testosterone levels

## Discussion

This study aimed to investigate the relationship between BMI, vitamin D, and testosterone levels in patients attending the outpatient clinic at Hayatabad Medical Complex, Peshawar, Pakistan. The findings revealed a significant inverse correlation between BMI and both vitamin D and testosterone levels, consistent with the results of multiple studies conducted in various populations worldwide.

Several studies have demonstrated that individuals with higher BMIs are more likely to have lower vitamin D levels, a finding supported by our study [7]. The underlying mechanism is thought to involve the sequestration of vitamin D in adipose tissue, reducing its bioavailability [8]. Wortman et al. proposed this hypothesis after finding that obese individuals had reduced cutaneous synthesis of vitamin D following sun exposure, leading to lower circulating vitamin D levels [9]. Our study is in complete agreement with this observation, as patients with obesity (BMI ≥ 30 kg/m^2^) showed a significant prevalence of vitamin D deficiency.

A meta-analysis by Vanlint in 2013 further confirmed the association between obesity and vitamin D deficiency, suggesting that for every unit increase in BMI, there is a corresponding decrease in serum vitamin D levels [10]. This finding is in line with our study’s correlation coefficient (r = -0.79), which indicates a strong inverse relationship between BMI and vitamin D. Similar patterns have been observed in studies conducted in both Western and South Asian populations, such as one by Vimaleswaran et al., indicating that this association is consistent across different ethnic groups [11].

Our study also found a negative correlation between BMI and testosterone levels (r = -0.87), which is consistent with previous research. Obesity is a well-known risk factor for low testosterone levels, particularly in men [12]. Kaplan et al. reported that men with higher BMIs had lower testosterone levels, which was attributed to increased aromatase activity in adipose tissue, leading to the conversion of testosterone to estrogen [13]. This mechanism explains the inverse relationship observed in our study, where obese patients exhibited significantly lower testosterone levels than those with normal BMI.

Some other literature has also linked low testosterone levels with metabolic syndrome, a condition frequently associated with obesity [14]. Low testosterone has been implicated in increased insulin resistance, altered fat distribution, and reduced muscle mass, all of which are characteristic of individuals with higher BMIs [15]. These findings are further corroborated by research from Traish et al., who highlighted the role of testosterone in metabolic regulation and the potential benefits of testosterone replacement therapy in obese men with low testosterone [16].

The positive correlation between vitamin D and testosterone levels observed in this study (r = 0.87) is supported by several investigations into the role of vitamin D in hormone regulation. Wehr et al. found that men with higher vitamin D levels also had higher testosterone levels, suggesting that vitamin D may play a role in modulating the hypothalamic-pituitary-gonadal axis [17]. This observation has been further supported by Pilz et al., who reported that vitamin D supplementation led to an increase in testosterone levels in men with vitamin D deficiency [18].

While the exact mechanisms remain unclear, it has been hypothesized that vitamin D may influence testosterone production by regulating calcium homeostasis, which is essential for testosterone synthesis [19]. Additionally, vitamin D receptors are expressed in Leydig cells, where testosterone is produced, suggesting a direct role of vitamin D in steroidogenesis [20]. Our findings align with these studies, suggesting that maintaining adequate vitamin D levels could potentially improve testosterone levels, particularly in individuals with low BMI or vitamin D deficiency.

Strengths and limitations

The strengths of this study include its relatively large sample size (n = 272) and the use of standardized methods for measuring BMI, vitamin D, and testosterone levels. This allowed for a robust analysis of the relationships between these variables. Furthermore, the study was conducted in a tertiary care hospital, providing access to a diverse population with varying BMIs and health statuses.

However, several limitations must be acknowledged. First, the cross-sectional design of the study limits the ability to establish causality between BMI, vitamin D, and testosterone levels. Longitudinal studies are required to determine whether changes in BMI directly influence changes in vitamin D and testosterone levels over time [21]. Second, while we controlled for known confounders such as age and sex, other factors such as dietary intake, sun exposure, and physical activity were not accounted for, all of which could affect vitamin D and testosterone levels [22]. Finally, the study population was limited to patients from a single hospital in Peshawar, which may limit the generalizability of the findings to other populations [23].

Implications for future research

Given the significant associations observed between BMI, vitamin D, and testosterone levels, future research should focus on longitudinal studies to further explore these relationships. In particular, interventions aimed at reducing BMI through weight loss or lifestyle modifications could be investigated to determine their effects on improving vitamin D and testosterone levels [24]. Additionally, randomized controlled trials examining the effects of vitamin D and testosterone supplementation in individuals with obesity and deficiency in these biomarkers could provide insights into potential therapeutic approaches [25].

Furthermore, research in different ethnic populations is warranted to confirm the findings observed in this study [26]. The interplay between BMI, vitamin D, and testosterone remains complex, and further exploration of the underlying biological mechanisms is needed [27]. Understanding these relationships could contribute to better management strategies for individuals with obesity and related hormonal imbalances [28]. Future studies may also examine the role of testosterone replacement therapy and vitamin D supplementation as adjunctive treatments for improving metabolic health in obese individuals [29].

## Conclusions

Body mass index is significantly associated with vitamin D and testosterone levels in this study. This study indicates that higher BMI ranging from overweight to obesity has an association with lower vitamin D and testosterone levels as compared to persons with a normal BMI. These findings suggest that addressing obesity could help mitigate hormonal imbalances, such as vitamin D deficiency and low testosterone, which are linked to metabolic health risks. This can also be hypothesized that obesity can be a risk factor for vitamin D and testosterone deficiency.
